# Subalpine woody vegetation in the Eastern Carpathians after release from agropastoral pressure

**DOI:** 10.1038/s41598-022-22248-3

**Published:** 2022-10-25

**Authors:** Józef Mitka, Stanisław Kucharzyk, Jorge Capelo, Alina Stachurska-Swakoń

**Affiliations:** 1 Faculty of Biology, Jagiellonian University Cracow, Kraków, Poland; 2 Ecological Education Unit, Bieszczady National Park, Ustrzyki Dolne, Poland; 3 National Institute of Agrarian and Veterinarian Research, Oeiras, Portugal; 4 ECOCHANGE, CIBIO-InBIO - Research Center in Biodiversity and Genetic Resources, University of Porto, Porto, Portugal

**Keywords:** Ecology, Ecology, Environmental sciences

## Abstract

The subalpine vegetation in the Eastern Carpathians has been under agropastoral influence as a high-mountain open pasture for about five centuries. Today, the subalpine zone released by human intervention is growing as thickets. In this study, we use a numerical model of tree crowns (CHM, Canopy Height Model) based on laser scanning (LiDAR) and a high-resolution digital terrain model (DTM) to delineate the subalpine thicket distribution. Anselin ‘Local Moran's I’ statistic was used to find hot and cold spots in vegetation cover. We used a logistic generalized linear model (GLM) and Principal Component Analysis (PCA) to set for the historical, climatic and terrain conditions candidates as the predictors of the present-day distribution of vegetation hot spots. We use variance partitioning to assess the interaction of climate and terrain variables. The resulting model suggests key environmental controls that underlie the vegetation pattern. Namely, snow in terrain depressions protects woody vegetation against abrasion and winter drought and increased insolation reduces the site humidity in the summer on S-E exposure hampering re-vegetation. In addition, the increasing distance from the treeline declines the rate of secondary succession. In all, the spatial model predicts the 35% coverage by thickets as a theoretical maximum of available climatic-terrain niches. The results suggest that the growth of the subalpine thicket, in the face of growing global temperature, may be restricted due to the limited number of niches available.

## Introduction

In the last two decades, the number of studies on Arctic and Alpine biomes has increased. Such zones, and timberline shifting, are sensitive indicators of global change, particularly of global warming^1–8^. However, the timberline position alone as the sole indicator is not applicable everywhere. In most mountain ranges in Europe, subalpine and alpine communities were used as high-mountain pastures for centuries, which usually led to a decline of the upper limit of the natural forest^9–14^.

Moreover, the rate of regeneration of forests and the speed of response due to climate change differ in various regions^15^. As expected, the tendency to increase the upper limit and the formation of ecotone systems is currently intensifying^16^. Nevertheless, in some cases, a different shift rate has been observed from what is expected from climate warming alone^17^. Therefore, we believe that in landscapes with a long history of human usage, other factors that determine the position of the timberline should be taken into account.

**Figure 1:**
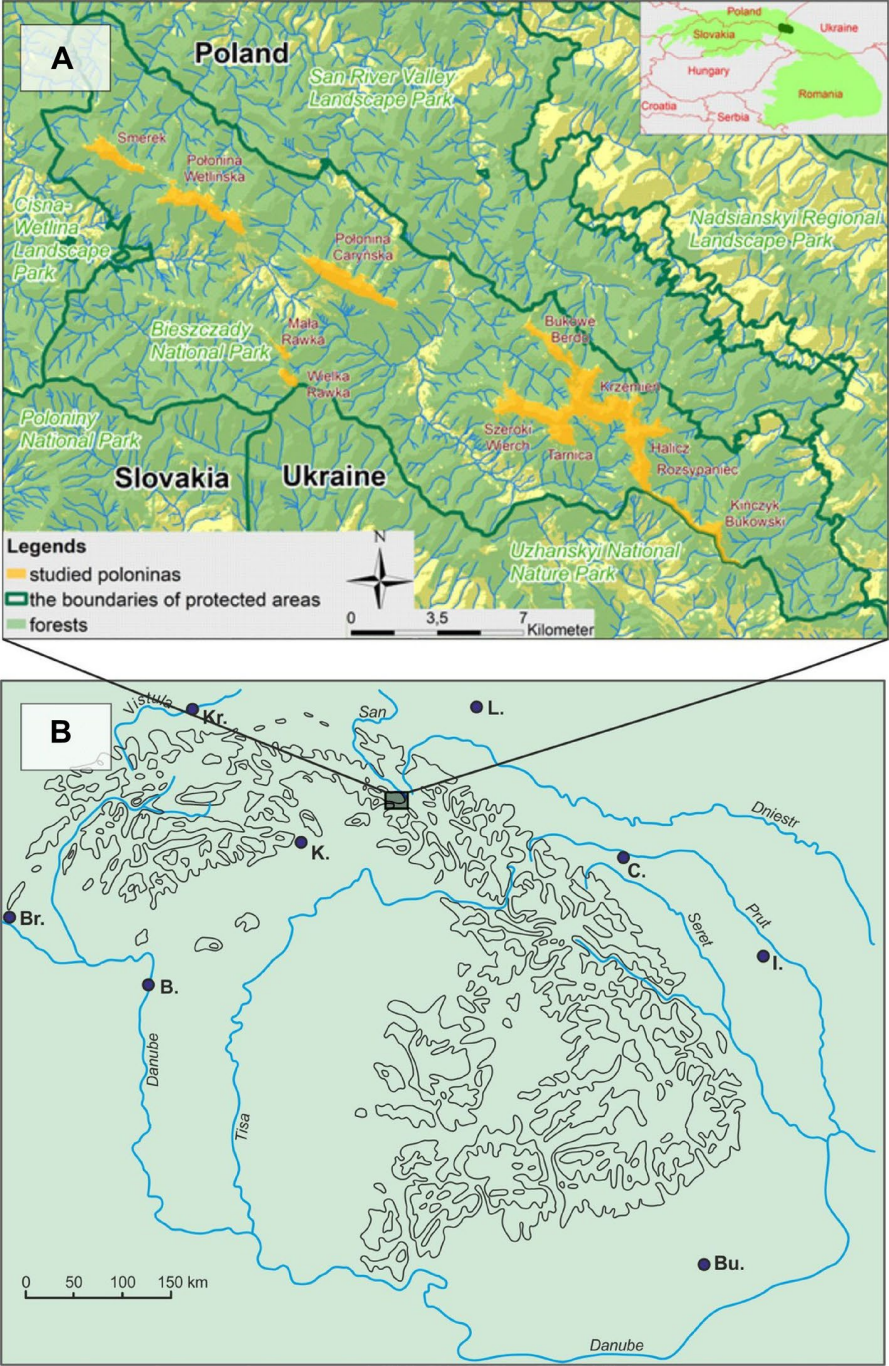
Map of the Bieszczady National Park (**A**) and its localization in the Carpathians (**B**): Br—Bratislava, Bu—Bucureşti, B—Budapest, C—Černivce, I—Iassy, K—Košice, Kr—Kraków, L—Lviv. Credit: Map generated with QGIS Version 3.20 (https://www.qgis.org/).

In the Western Bieszczady (Polish Eastern Carpathians), the progressive succession process after grazing abandonment encompasses several vegetation types. A subalpine meadow called *polonina* is adjacent to a broadleaf, sycamore-beech forest (Dentario glandulosae-Fagetum), and not directly in catenal contact with the spruce forest (of *Picea abies*) as in most of Europe^14,18–20^. (Fig. 1, Supplementary information Figs. S1 and S2). Although it was abandoned 80 years ago, the establishment of beech and other trees in the subalpine meadow is still limited^21,22^.

There is evidence that past human land use lowered the natural upper forest limit in the Eastern Carpathians^23–28^. In fact, both wild and domestic large herbivore ungulates contributed to the expansion of species-rich *polonina* meadows (Fig. 6A). This raises the question of what the size of subalpine grasslands would be under strict ‘natural conditions’^29^, comparing to its actual extent and that was the result of cattle grazing and farming history. Today, these semi-zoo-anthropic grasslands form the typical traditional farming landscape of the Eastern Carpathians and the Southern Carpathians, extensively economically used until the second half of the twentieth century^13^.

The area studied has well-described elevational gradient of flora and vegetation, and a well-known history of anthropogenic influences^24,26,30–32^.

The objective of this study is to determine the effect of topographic-climatic variables and human agropastoral activity on the present spatial distribution of the subalpine thicket in the Bieszczady National Park (Western Bieszczady Mountains, Polish Eastern Carpathians). The variables come from laser scanning (LiDAR) and digital terrain (DTM) models. We used a Generalized Linear Model (GLM) to approach the major ecological drivers of current successional processes that act near the timberline. Furthermore, in a region released from direct human pressure, we intended to estimate the climatic-terrain microtopographic niches where the future growth of subalpine vegetation may be expected.

## Materials and methods

### Study area

The forest near its upper limit is either dominated by beech (*Fagus sylvatica*) alone or co-dominated by sycamore (*Acer pseudoplatanus*) and occurs up to an altitude of 1000–1260 m a.s.l. (Supplementary information, Fig. S2). The subalpine meadow (called here *polonina*, Ukr. "open site") is situated above the forest border and partially overgrown by thickets. They are formed by rowan (*Sorbus aucuparia*), willows (mainly *Salix silesiaca*), green alder (*Alnus viridis*), and sycamore, which form specific subalpine plant associations^26^. The thickets form a transition stage in succession toward potential Medio-European subalpine beech woods with *Acer*—Aceri-Fagetum (https://eunis.eea.europa.eu/habitats/10188), which in the Bieszczady Mts maintain, for the most, a natural character^30,49^.

The succession stage of beech is limited only to a narrow zone at the upper forest limit^19,21,34,35^. Generally, the upper forest limit forms a diffuse treeline with ‘krummholz’ of shrubby thwarted tree individuals that survive only in a few microsites (spatial heterogeneity). The occurrence of woody plants is, in general, controlled by spatial stochasticity and temporal heterogeneity^50^. Individuals or small groups of European spruce (*Picea abies*) are scarce in mountain pastures^19^.

The climate parameters in the mountain meadows span an average annual air temperature of 2 to 3 °C (− 6.7 °C in January, and 11.5 °C in July) and an average rainfall often exceeding 1200 mm yr^−1^. In the autumn, winter and spring months, strong (10.8–13.8 m/s) and very strong winds (13.9–17.1 m/s) are often felt. The highest wind speeds (on average 5 m/s) occur with the air masses coming from the southern direction (about 34% of observations). The average number of days with snow cover per year in the higher parts of Bieszczady National Park is estimated to be 150–155 and is, in some years,180 days^51,52^.

The study area belongs to the so-called Central Carpathian Depression. Its lithology consists of folded Tertiary facies composed, in near-surface parts, of sandstone-shaly and siltstone Krosno beds of Oligocene age. The *poloninas* often occur in thick-bedded sandstone complexes. The morphology of this region is structurally controlled and characterized by a rectangular pattern of ridges linked with low passes, forming a "grate" arrangement (see Fig. 1A). Ridges are characterized by relatively long slopes that show singular or composite breaks. Elevation differences between the highest ridges (Tarnica Mt 1346 m) and main bottom valleys range between 400 and 500 m, attaining locally 600 m^53,54^.

The morphology of the Bieszczady Mts follows their geology. Almost all the tops are found on resistant layers of the Carpathian flysch, whereas the lower parts are built on low-resistant bedrock. As to soils, in the subalpine zone, shallow and skeletal rankers develop in cool and humid climate up to 50 cm in depth^55–57^.

Information on historical land use is given in Supplementary Information.

### Data preparation and spatial analysis

**Figure 2:**
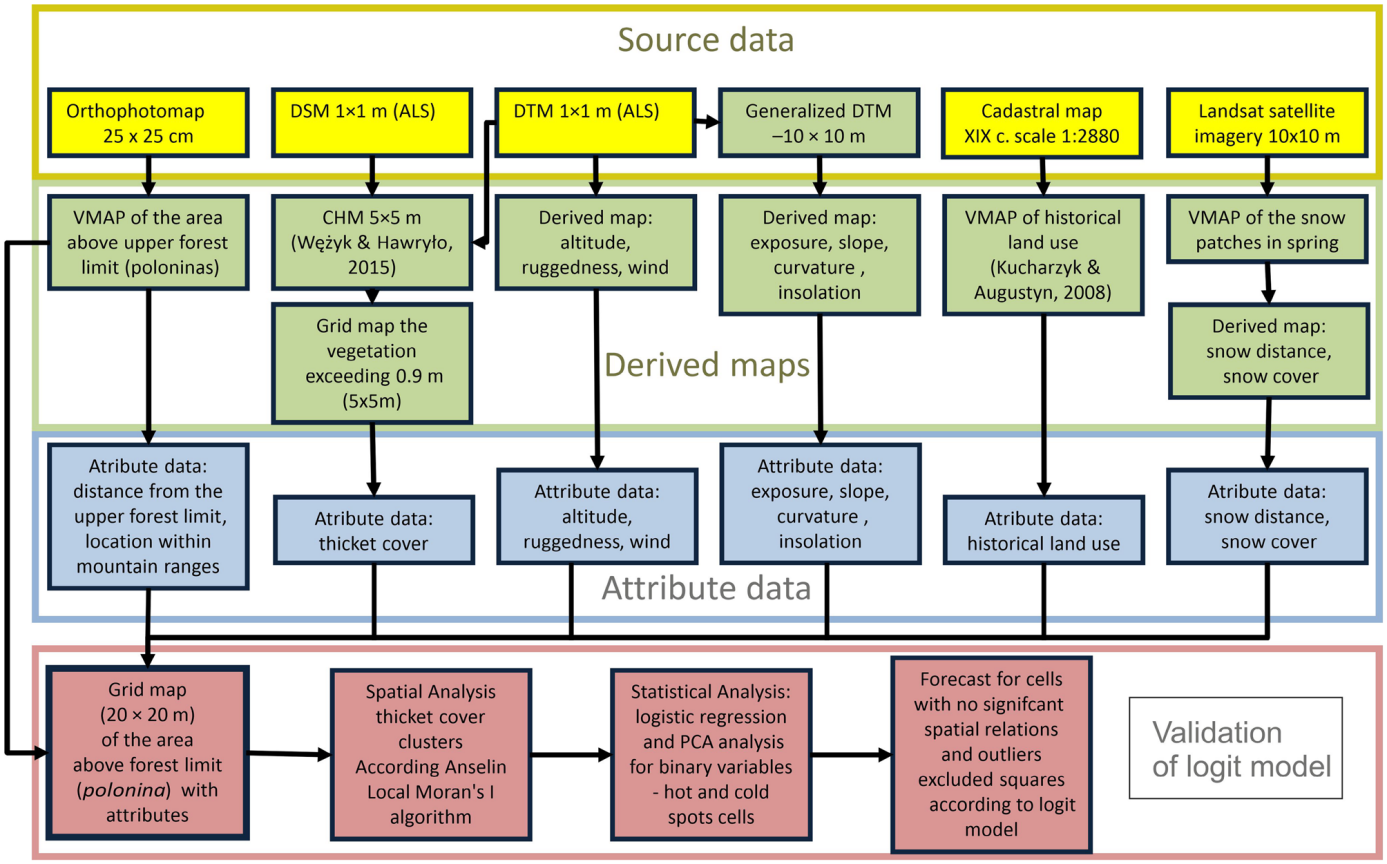
Flow diagram of the data handling. For details see Table 1.

The flow of data treatment is shown in Fig. 2. We used 12 variables (10 continuous and two categorical) that are expected to affect the distribution of subalpine thicket patches (Table 1). They were characterized as to the location with respect to human settlements, past land use (both categorical), distance from the contemporary upper forest border, snow cover and other climatic related variables, and parameters determining the topographic diversity of the terrain, according to the high-resolution DTM^21,22,26,32–35^.

**Table 1:** List of driving factors in this study.

Category	Data	Variable	Unit	Min. to Max. value	Type of variable	Source/grid cell size
**Terrain conditions**						
Geography	Altitude a.s.l	*Altitude*	m asl	980 to 1345	Continuous	DTM 1 × 1 m
Geography	Azimuth	*Exposure*	°	0 to 180	Continuous	DTM 10 × 10 m
Geography	Slope angle	*Slope*	°	1 to 45	Continuous	DTM 10 × 10 m
Topography	Profile Curvature	*Curvature*	–	− 5.512 to 3.968	Continuous	DTM 10 × 10 m
Topography	Terrain Ruggedness Index TRI	*Ruggedness*	m	0.152 to 2.722	Continuous	DTM 1 × 1 m
Spatial	Distance from the forest line	*Distance*	ln(m)	0 to 6.18	Continuous	Orthophotomap 25 × 25 cm
**Historical**						
Historical	Historical land use	*Land use*	–	1 to 3^a^	Categorical	–
Historical	Degree of human impact	*Location*	–	1 to 3^b^	Categorical	–
**Climate**						
Climate	Insolation index	*Insolation*	kWh m^2^	549 to 1319	Continuous	DTM 10 × 10 m
Climate	Leeward index	*Wind*	–	0.7747 to 0.3554	Continuous	DTM 1 × 1 m
Climate	Snow patches cover	*Snow cover*	%	0 to 100	Percentage continuous	Satellite imagery 10 × 10 m
Climate	Snow patches margin distance	*Snow distance*	m	− 80 to 320	Continuous	Satellite imagery 10 × 10 m

^a^1—pastures, 2—partially wooded meadows, 3—meadows.

^b^1—Smerek, Połonina Caryńska, Połonina Wetlińska, 2—Tarnica&Halicz, 3—Mała&Wielka Rawka.

For the analysis of the contemporary distribution of subalpine thicket in the Bieszczady Mts, we used data from laser scanning (LiDAR) performed in the Bieszczady National Park in 2015 and its conversion to a numerical model of tree crowns (CHM, canopy height model with a resolution of 5 × 5 m—index: 95th percentile; see^36^. In the CHM, the differentiation of vegetation height is estimated by the difference between the high resolution (1 × 1 m) numerical land cover model and the numerical terrain model (DTM). Data on the area above the upper border of beech forests were obtained from the photo interpretation of a 2015 orthophoto map. The area of meadows thus obtained was divided into a 20 × 20 m grid, selecting 43,077 squares entirely located above the upper forest limit (1723.08 ha). In each grid there were 16 squares 5 × 5 m. For them, according to CHM, thicket cover was assessed (on a scale from 0 to 16) based on the presence of vegetation exceeding 0.9 m in height.

Furthermore, a clustered spatial distribution of subalpine thickets was tested using Moran I statistics (ArcMap spatial package). Further on, clear-cut clusters, those with high and low values (hot spots and cold spots—binary response variable) and groups of outliers were defined according to the Anselin Local Moran's I algorithm^37,38^. The clusters with high and low vegetation cover values (hot and cold spots—binary response variable) were then regressed with the 12 explanatory variables (Table 1)^39^ in the GLM.

Terrain and climatic variables (predictors) were defined as mean values for individual grid squares. Calculations of the average *Exposure* and *Slope* values were performed in ArcMap 10.2 with the Spatial Analyst extension (zone statistics) and in the analogous QGIS function (version 3.14.1-Pi). The *Insolation* index (supply of solar energy) was calculated using the algorithm of Pierce and co-authors^40^ implemented in the Spatial Analyst extension (ArcMap 10.2). Absolute *Altitude* (m a.s.l.), *Exposure* (azimuth), *Slope*, TRI—topographic *Ruggedness* index predictor^41^, *Exposure, Insolation,* Profile *Curvature,* and Leeward Index (*Wind,* shows the "shade effect" of winds on the leeward slopes), a dimensionless index described by Böhner and Antonić^42^, were obtained from the DTM. Analyzes were performed with the algorithm implemented in SAGA (System for Automated Geoscientific Analyses) version 2.3.2.

*Distance* from the upper forest limit to subalpine thickets was transformed to its natural logarithm in the QGIS application (distance to the nearest hub). *Snow cover* was obtained by reclassification of satellite imagery from Sentinel-2 (L2A) on 2 April 2017 and TCI—True Color Image (TCI) –2A_T34UFV_20170402T093031_TCI_10m, available at https://scihub.copernicus.eu/. The analysis of the behaviour of snow patches in spring is a surrogate for the inaccessible data on the variation in the depth of snow cover, as the more thick patches last longer. They were analyzed by calculating two parameters: the percentage of the area occupied in the grid square by the *Snow cover* and the distance of the square centroids from the snow patches (*Snow distance*). The distance of individual squares from the edges of the snow sheets was assessed with the ArcMap program (buffer analysis).

*Land use* categories were established according to a historical cadastral map from the mid-nineteenth century^22^. It encompassed the following scheme: 1—pasture, 2—partially wooded meadow, 3—meadow.

The *Location* within different mountain ranges was ordered according to the gradient of human anthropogenic influence related to the distance from the settlements: the highest influence (rank 1) in the region of Smerek—Połonina Caryńska—Połonina Wetlińska, a moderate (2) in Tarnica and Halicz and range, and the smallest (3) in Mała and Wielka Rawka region. A list summarizing all explanatory variables used in this study is given in Table 1.

### Identification of potential habitats for further succession (forecast)

The analysis of clusters not covered by logistic modelling (NN not statistically significant autocorrelation, LH low–high, geographical units with low values surrounded by geographical units with high values and HL high–low, geographical units with high values surrounded by geographical units with low values) can be treated as a prediction of the thicket development in areas where succession is today in *statu nascendi*.

### Data analysis

To evaluate correlations among variables, a PCA was performed with package *vegan*^43^. A binary logistic regression (LR, logit link^44^) on a set of explanatory variables (Table 1) to predict the appearance of thickets was performed with *Gretl 2020e* statistical package^45^. The explanation variable based on CHM 5 × 5 matrix cells belonging to clusters with low (LL) and high thicket cover values (HH). It should be noted that GLM is adequate for mixed heterogeneous data, such as ours, because it does not have many of the critical assumptions of linear regression, especially regarding linearity, normality and homoscedasticity^46^. The Wald test for the logistic model was performed with "wald.test" in the *aod* package. The validity of the GLM model was verified by a k-fold cross-validation with the *modeler*. The relative importance of predictors in a binomial logistic GLM^47^ was calculated with *dominanceanalysis* package^43^.

The explanatory data set was divided into subsets of variables: terrain data (*Altitude, Distance, Exposure, Slope, and Ruggedness*), climatic (*Insolation, Wind, Snow cover, Snow distance*), and historical (*Land use, Location*). The nominal (qualitative) response variables (*Location* and *Land use*) were coded as factor variables (0/1 dummy). To evaluate how much variance is a priori explained by each of the subsets and their interactions, a variance partitioning procedure based on RDA, as described by Borcard, Legendre & Drapeau^48^, was carried out with "varpart" procedure in *vegan*^43^.

## Results

**Figure 3:**
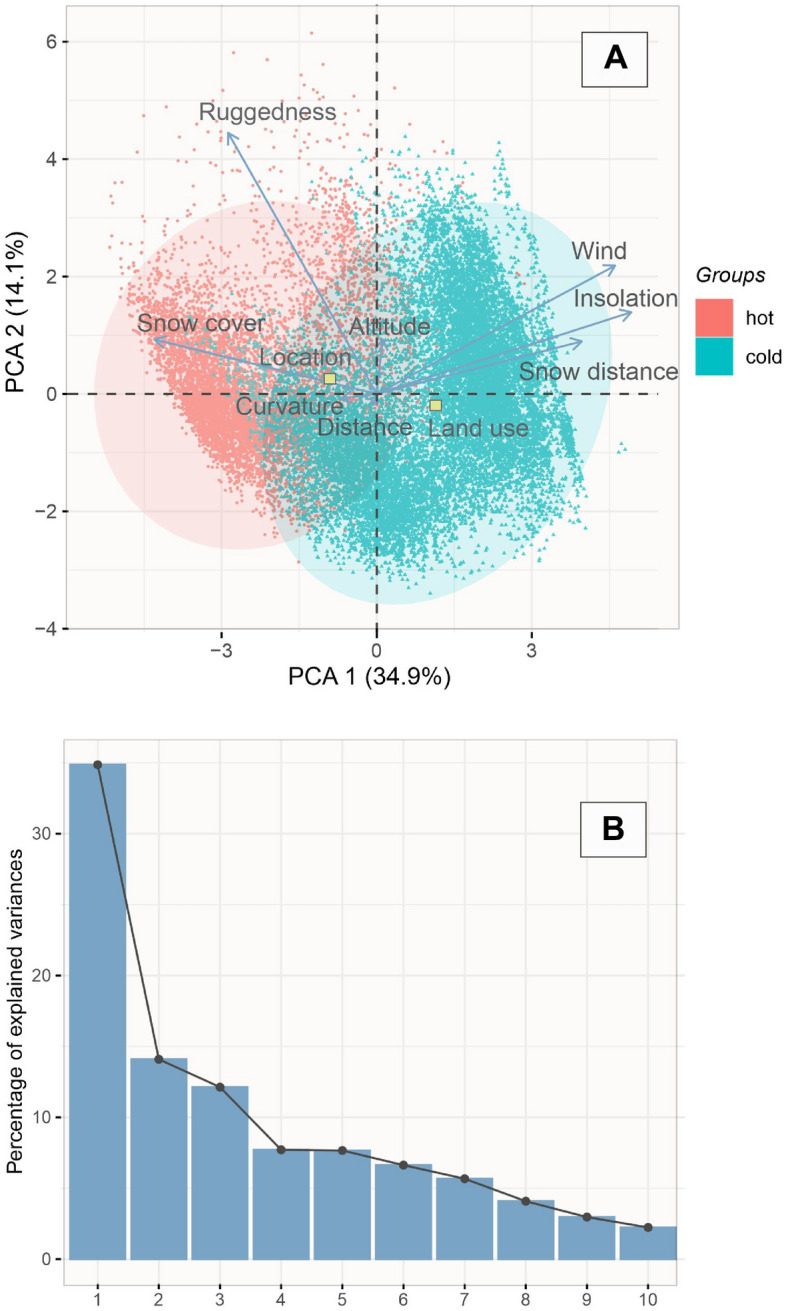
(**A**) Principal Component Analysis (PCA) of 20,089 hot (HH) and cold (LL) plots (NN and outliers excluded, see Fig. 4). (**B**) Scree plot. The percentages in axis titles stand for percentages of explained variation of the respective axis. Ovals denote 95% confidence intervals. *Land use* and *Location* are dummy variables. Variables are explained in Table 1. Credit: R Core Team^43^.

The first two axes of the PCA explain 49% of the total variability (Fig. 3). The first axis (34.9%) is negatively loaded with *Snow cover* and *Ruggedness,* and positively loaded with *Insolation, Wind,* and *Snow distance.* The second axis (14.1%) is positively loaded with *Ruggedness.* The remaining variables are located near the origin of the coordinate system and, thus, are insignificant.

The results of the Moran I global statistics from the ArcMap package indicate a statistically significant spatial autocorrelation of the thicket cover in squares (Moran index: 0.656, standardized Moran I statistic z score: 734.03 with a significance level lower than 0.001, default threshold distance: 102 m, inverse Euclidean distance).

**Figure 4:**
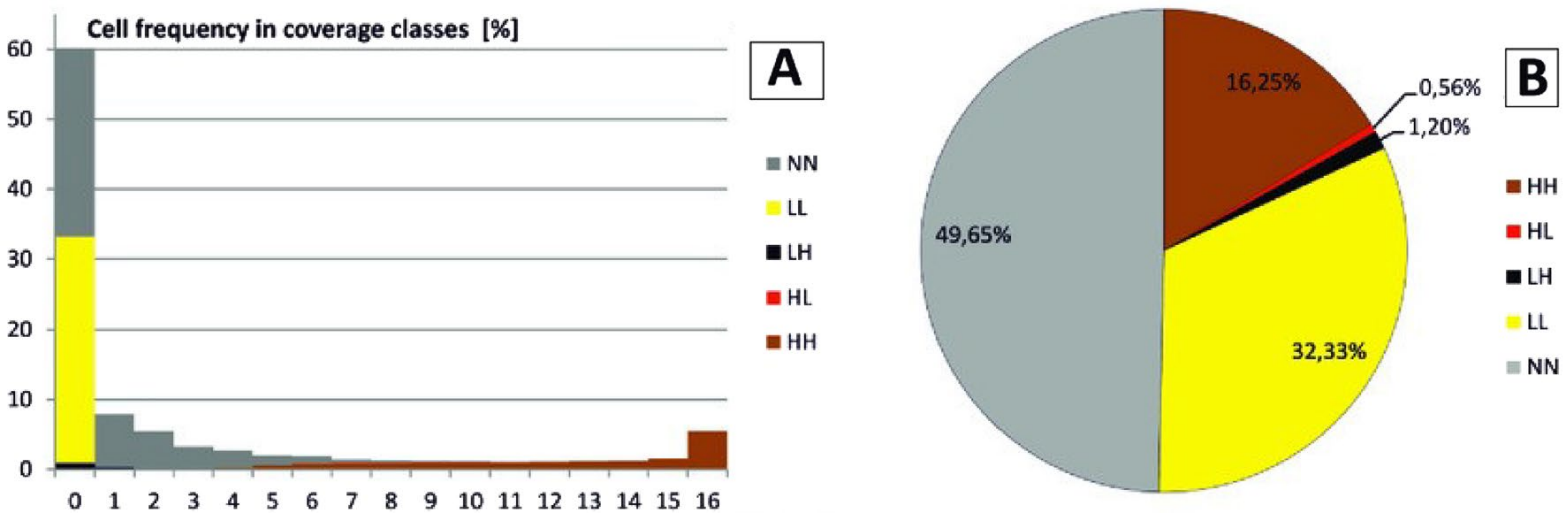
(**A**) Distribution of the frequency of grid cells (20 × 20 m) depending on the scrub coverage in 16 cover-classes; (B) The percentage of cluster type according to the Anselin Local Moran's I statistic. HH—cell clusters with high coverage (hot spots), LL—cell clusters without scrub or low coverage values (cold spots), HL—cells with high values, outliers from the background with low values, LH—cells with low values, outliers from the background with high values (both statistically significant spatial outliers), NN—cells with no significant spatial relations. For the details of the calculations see Supplementary Information—Appendix. Credit: ArcMap software.

**Figure 5:**
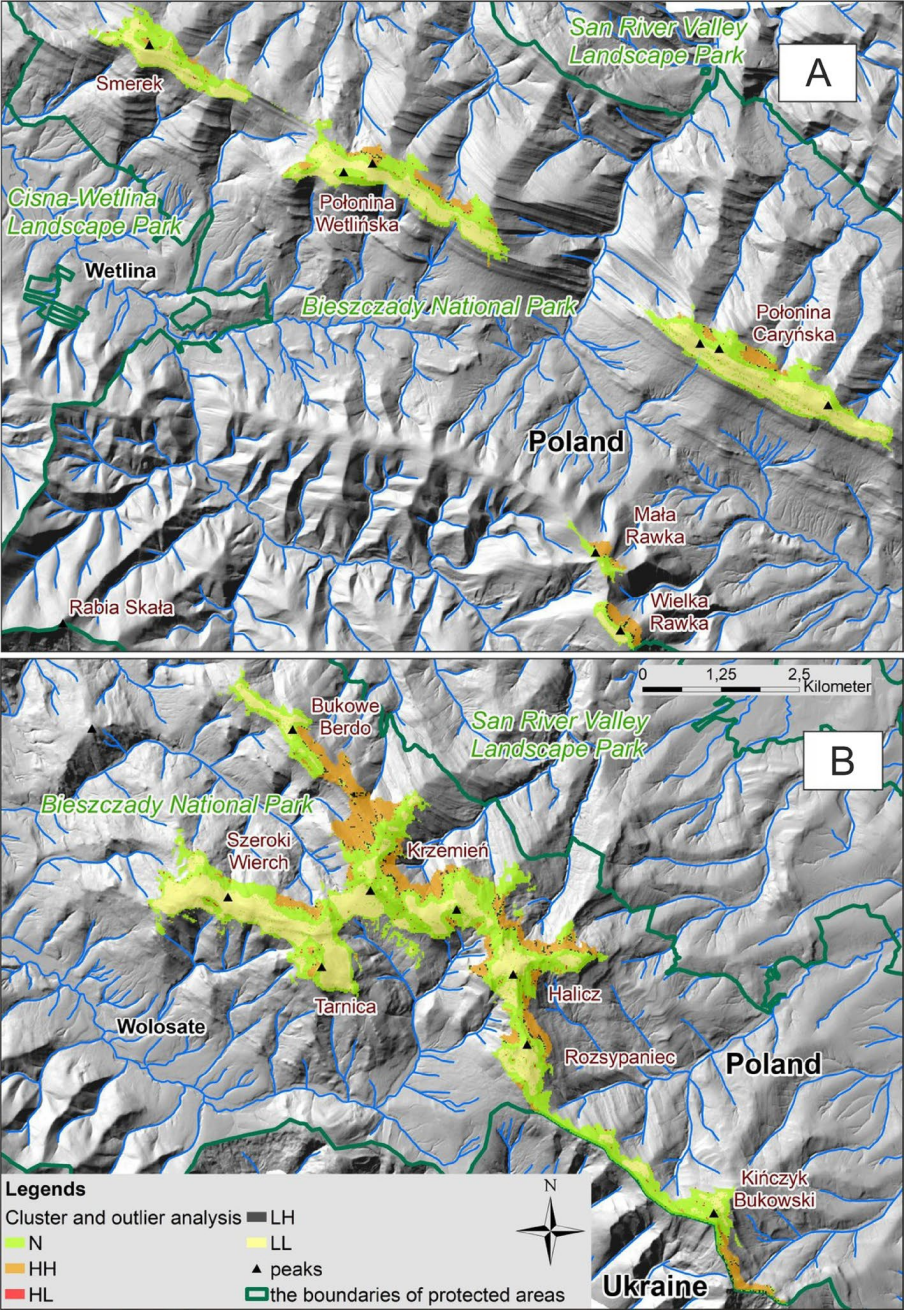
Distribution of cells depending on the classification of clusters according to the Anselin Local Moran's I statistic. (**A**) Part N-W of the Bieszczady National Park; (**B**) part S-E. Cluster types as in Fig. 4. Credit: Map generated with QGIS Version 3.20 (https://www.qgis.org/).

The spatial distribution of clusters with cold LL and hot spots HH and the remaining categories of cells is shown in Fig. 4A,B. According to data from the numerical model of tree crowns (CHM), the current global cover of subalpine thickets is 20.4% of the mountain meadows. The distribution of the square frequency in the cover classes suggests that up to 60.2% of the mountain meadows lack shrub vegetation and in the remaining 19.4% the thickets occurred on the cover scale 1–4 (Fig. 4A). Around 50% of the cells did not show statistically significant autocorrelation (NN category, Fig. 4B). Their distribution in the Bieszczady National Park is shown in Fig. 5.

The logistic GLM (logit model) of the distribution of clusters with high (hot) and low (cold) thicket cover values (a binary variable, Table 2) explains 54.2% of the total variance (McFadden pseudo R = 0.612) with an accuracy of 'proper predictions' = 90.3%. Overall Wald test = 23.6, *p* < 0.001. The K-fold cross-validation of the logit model returned Accuracy = 0.896 and Kappa = 0.763. Positive estimates of the GLM coefficients corresponding to the variables: *Snow cover, Ruggedness,* and *Location.* The importance of *Land use* and *Location* points to the relatively intact high mountain vegetation on Mała Rawka ridge. It is the most remote, and the least changed by human agropastoral activity, range in the Western Bieszczady Mts. An univariate model conditional dominance analysis pointed to the greatest importance of *Ruggedness* and *Distance*, and general dominance to *Ruggedness,* followed by *Insolation* and *Distance* (Supplementary Information Fig. S3A, B).

**Table 2:** The results of the logistic GLM estimation for clusters with high and low values (binary variables, hot and cold spots).

Variable	Estimate	Stand. Error	*z*	Marginal effect^1^	Wald test^2^
Constant	14.928	0.793	18.83	–	123.2
*Altitude*	− 0.006	0.0008	− 7.398	− 0.0008***	175.0
*Curvature*	− 0.336	0.048	− 7.056	− 0.045***	195.1
*Ruggedness*	3.895	0.105	37.21	0.526***	201.4
*Distance*	− 1.290	0.049	− 26.47	− 0.174***	956.8
*Land use 2*	− 0.375	0.279	− 1.341	− 0.044 n.s	45.6
*Land use 3*	− 1.570	0.088	− 17.73	− 0.153***	263.2
*Location 2*	3.876	0.197	19.66	0.746***	23.6
*Location 3*	2.060	0.085	24.20	0.237***	956.8
*Insolation*	− 5.746e−06	3.43E−07	− 16.77	− 7.756e−07***	137.8
*Wind*	− 1.642	0.234	− 7.026	− 0.222***	175.0
*Snow cover*	0.0123	0.0008	14.47	0.002***	195.0
*Snow distance*	− 0.009	0.001	− 8.009	− 0.001***	124.6

Two variables were eliminated from the model: Exposure and Slope due to high collinearity with Ruggedness and Insolation. For the same reason, the categorical variables Land use 1 and Location 1 were automatically removed. For the description of the variables, see Table 1.

^1^***p < 0.001, n.s.—not significant.

^2^All values at *p* < 0.001.

The NN class, where no statistically significant shrub patches were found, now occupies 49.65% of the meadow area (Fig. 4B). According to the logit model, potentially favorable conditions for the development of shrub communities occur in 1/3 of the cells representing this class (see Supplementary Information, Table 1). The HL and LH classes represent rare and exceptional cases related to the presence of clumps of less common species (e.g., spruce clusters) or specific habitat conditions; hence, in this case, we assumed that the prediction is uncertain and poorly determined by the habitat conditions specified in the model. Therefore, the HL and LH classes were excluded from the vegetation predictive model.

**Figure 6:**
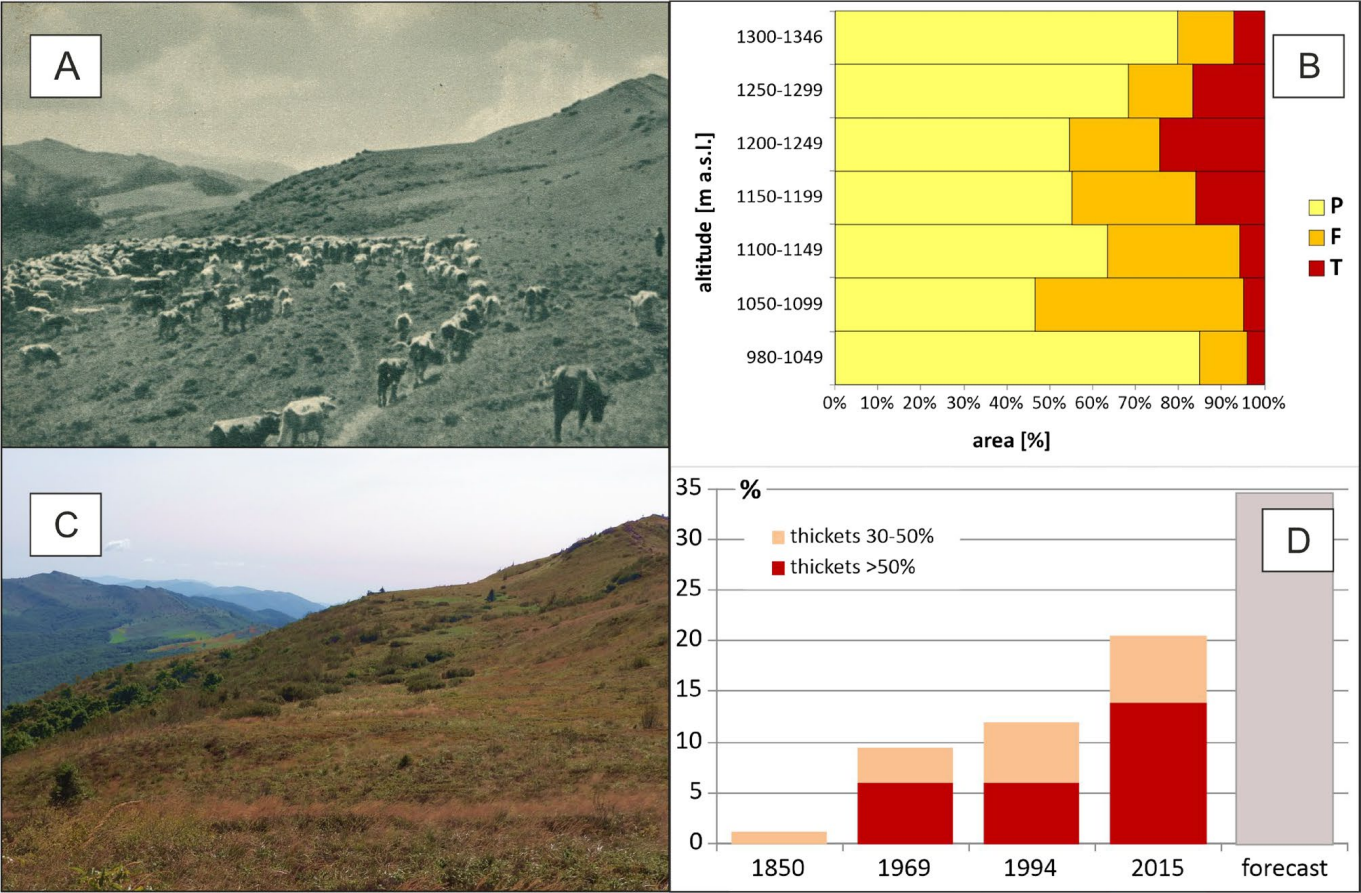
The present and historical extent of subalpine thicket and expected growth. (**A**) Vegetation cover in Rozsypaniec ridge in 1930’ Photo credit: Public domain, source: https://polona.pl/item/widok-na-halicz-w-powiecie-turczanskim-1335-m-n-p-m,Njg2NjY1NTY/0/ #info:metadata; (B) vegetation types in altitudinal limits in 2015: open meadow (polonina)—P; subalpine thicket (present)—T, subalpine thickets (forecast)—F. See also Supplementary Information—Appendix. (**C**) Vegetation cover at present (2015), photo credit: S. Kucharzyk; (**D**) Dynamics of thicket cover. *Source*: 1850 (Kucharzyk & Augustyn, 2008), 1969 (Kucharzyk, pers. data), 1994 (Kucharzyk & Augustyn, 2008); 2015—according to logit model, and forecast—maximum available niches of thickests calculated according to logit model (see Supplementary Information).

Taking into account the model in its final form, the cover of compact thickets is expected to increase from about 20 to about 35% (Fig. 6D), mainly within 1050–1249 m (Fig. 6B). Historical and present photographs (Fig. 6A,C) show the moderate tempo of the thicket succession in Bieszczady's subalpine *polonina*.

We note that the model obtained (in hot and cold spots area) yields a prediction nearly 90% consistent with the present-day field observations. The few relevant underestimation errors are grouped in a small valley between Krzemień and Bukowe Berdo, where pastoral activity ceased the earliest, which the cadastral maps did not show. Re-evaluation errors are scattered randomly throughout the analyzed area.

**Figure 7:**
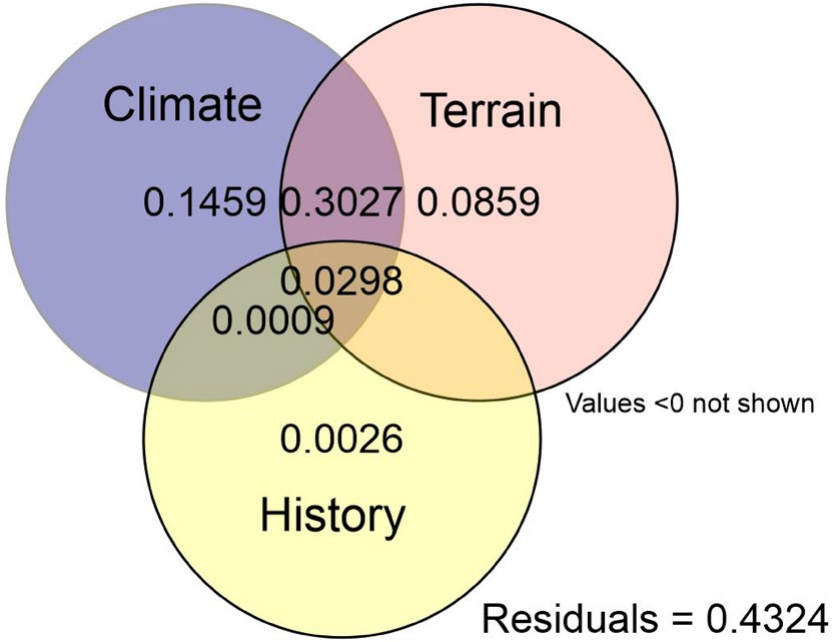
Venn diagram of variance partioning of data grouped into three subsets: Climate, Terrain, and History (see Table 1). Credit: R Core Team^43^.

The variance partitioning showed the importance of climate and terrain factors for the development of vegetation in the mountains. Together, they explain 30.3% of the total variation (14.6 and 8.6% alone, respectively). Historical factors contribute less than 1% (Fig. 7).

## Discussion

Shrub overgrowth in alpine pastures after the cessation of economic use has been observed, for several decades, in many European mountain systems^7,11,14,20,58–60^. In the Bieszczady Mts, the rate of colonization of subalpine pastures by woody species was also attributed to global climate change^34,35^. However, climate change in the Carpathians at locations higher than 1200 m is slower than at lower altitudes^61^. The present analysis shows that shrub thicket growth will be the most dynamic at lower elevations (1050–1249 m) and will be moderate at higher elevations (1250–1346 m). The prognosis is in line with Winnicki^26^ that the upper forest limit is naturally stabilized at 1250 m. In short, most present thicket formation in the upper parts should be, otherwise, due to successional recovery of woody vegetation over pasture and not so much by the rise of bioclimate/vegetation belts due to climate change.

The Western Bieszczady Mts outstand because the regeneration process was initiated as early as the mid-twentieth century and was not directly caused by economic cycles, but forced evictions of the local inhabitants instead as the effect of political decisions in 1946–1950^22,32^. Analysis of historical remote sensing materials^22^ shows succession towards thicket in the subalpine zone in the first two decades after the discontinuation of land use (1969 in Fig. 6D and Supplementary Information). Despite some pieces of evidence, some cautionary view on data over time must be adopted. First, several tree species, with distinct ecological behaviour, are involved, variations of vegetation physiognomy alone could result from distinct successional processes driven by somewhat distinct environmental drivers. For instance, there is evidence that not until 1994 changes in the range of pioneer communities with rowan (*Sorbus aucuparia*) and green alder (*Alnus viridis*) were significant (see Fig. 6D and Supplementary Information). Second, another apparent two-fold increase in shrub area occurred in 2015. This sharp change may be partially due to differences in the quality of the compared data, as the data from 1969 to 1994 come from the visual photo-interpretation of panchromatic or colour-infrared (CIR) materials^22^. However, the size of the observed change excludes the sole influence of methodological issues. Thus, the increase in thicket cover in recent years seems the natural process linked with the releasing of the high-mountain vegetation from the human direct influence.

Interestingly, there are no significant differences in the shrub area between 1969 and 1994 (Fig. 6D). By contrast, Durak et al.^34^ noted two cohorts of species variation from 1970–1974 to 1980–1994 on Mała Rawka. Indeed, by limiting the analysis to photogrammetric materials (data not shown), it is concluded that in the region of Mała Rawka a significant development of thickets occurred after 1969 (data not shown). However, it seems a unique phenomenon compared to the entire area of subalpine pastures in the Bieszczady Mts. The study by Durak et al.^34^ was carried out on the local scale (some percent of *polonina*). Our studies on the landscape scale significantly disagree with the overall conclusion of the authors mentioned above.

In the case of the Bieszczady subalpine mountain zone, the influence of dry southern winds blowing from the Pannonian Basin has been discussed for a long time^22,23,26,34,62^. It is believed that they prevent the development of spruce and lower the upper forest limit in the region^62^. We support the view that winter winds have significance by reducing the snow cover thickness^26,51^. We found a compound effect of snow-related predictors (*Snow cover*, *Snow distance*) and terrain *Ruggedness* that together explain 30% of the total variance of vegetation hot spots. The correlation and variance partition of the climatic and terrain characteristics point to multiple mutually enhanced interactions. Snow cover in terrain depressions protects trees from abrasion and winter drought and maintains moisture at the beginning of the growing season^63–65^. Drying winds hamper the growth of subalpine thickets, especially on the southern exposure (for the opposite, see^34^).

Durak et al.^34^ while examining the age of rowan on Wielka Rawka found that the expansion of the species was positively related to thin snow cover and low temperatures. They claimed that harsh winters without snow damage blueberry stands (*Vaccinium myrtillus)* and limit competition between species. In this effect, rowan germination is easier^34^. It is a biologically oriented hypothesis. However, the result may be interpreted differently, as unfavourable weather conditions could not influence the intensification of rowan recruitment but simply rejuvenate rowan tufts, damaging old shoots and stimulating young growth. It is widely believed that rowan is resistant to drought and frost^34,66–68^, and the combination of climatic, environmental and biological factors could be responsible for the ecological success of the species^4,66,67^*.* Specifically, the desiccation of vertical leaders that extend above the snow surface seems to be one of the key components of treeline advance^69,70^. The influence of winds, discussed above, was investigated using the Leeward index (*Wind*). Vegetation hot spots preferred the leeward north slopes. Observation of the deformation of trees and shrubs growing on the ridges in southern slopes shows that they often form the so-called flagship (krummholz) forms, with the direction of the "flags" pointing to the north. Therefore, the main factor does not appear to be the drying effect of the southern winds during the vegetation season, but the abrasion caused by the wind action and physiological drought stress in winters^65,71^.

In the present study, the most important predictors of the rowan thicket distribution turned out to be indices that are complex derivatives of DTM, especially *Ruggedness* (TRI) associated with geomorphological diversity: irregularities in relief in the form of rocks, sinkholes, and boulders. A characteristic feature of the morphology of the ridges and slopes in the Bieszczady Mts, especially in higher positions, is the presence of ridges, tectonic breaks, and rock outcrops, at the foot of which there was deposited rock debris (*grechot* in Polish) of periglacial origin^26,55,73^. Such topographic irregularities are shelters against unfavorable environmental conditions and form the regeneration niche for seedling recruitment and growth (Supplementary Information Fig. S4)^74–76^. Trusches (*Turdidae*) use protruding rocks on open slopes eating rowan fruits, which favours sowing seeds^35,77,78^. In addition, insolation was significant, positively correlated with the occurrence of cold spot clusters, and pointed to a negative influence on the growth of subalpine thicket.

Higher insolation in the mountains prolongs the vegetation season, which most often increases biomass. Still, it reduces the humidity of the habitat in the summer^79,80^. It is an important factor on the southern-eastern slopes, where it could lead to drought conditions in summer. The importance of insolation as a key factor in reducing thicket stands and affecting soil processes in the mountains was indicated in earlier studies^81–83^.

In the Bieszczady Mts, the least insolated places, preferred by the subalpine thicket, are concentrated on the northern inclined slopes. They are sheltered from the southern winds. Interestingly, similar relationships were found in abandoned pastures on the north slopes with rowan and green alder in the southern Alps. The similarities to the Bieszczady thickets in physiognomy, species composition, and topographic conditions of occurrence are striking, although the climate here is much milder^14^. In addition, in the monsoon climate of the eastern Tibetan Plateau in high mountain situations (above 4000 m), where annual precipitations are low (450 mm), more trees are planted on the north-facing slope above the present treeline, indicating a more favorable regeneration condition, compared to the east-facing slope^84^. Here, *Picea likiangensis* var. *rubra* (Balfour spruce) attains ca. 40 cm of height in the eastern slope, compared to an average 3.2 m (rowan, green alder) on the favorable N-W slope in the Bieszczady Mts.

Among other important predictors not directly related to topography, a relevant spatial characteristic (not calculated from the DTM) is the *Distance* from the upper forest limit. Through the side protection against wind and sunlight, the forest's edge favors the development of thickets^19,22,32^. Additionally, at the edge of the forest, there is a concentration of snow blown from the subalpine meadows^52^.

## Conclusions

Although the generalized logistic linear regression model (GLM) explains 54.2% of the total variance, it nevertheless points to significant determinants of further shrub succession in the subalpine region. The variance partitioning showed that climate and terrain account for 30% of the total variability, pointing to its mutual interdependence. *Snow cover, Insolation,* and *Ruggedness* efficiently explain the spatial distribution of the subalpine thicket in the Eastern Carpathians. Topographic irregularities (stone debris, local hollows) create shelter spots with prolonged snow cover protect vegetation against the wind abrasion in winters and physiological drought in springs. Topography and climate, mutually interrelated, are the driving factors that enable the regrowth of the subalpine thicket. The GLM predicts that the subalpine thicket will stabilize at 1050–1250 m with a cover of approximately 40–50%. Thus, the highest parts (above 1250 m) of the Eastern Carpathian will remain open in 70–80%. In this way, high-mountain vegetation with endemic Carpathian plant species will stabilize. The role of terrain characteristics, especially *Ruggedness,* invokes the problem of future vegetation changes in the face of growing global temperature. The limited number of climatic-terrain niches for the growth of rowan thickets (*Sorbus aucuparia*) in the Bieszczady Mts may limit the direct influence of milder climate on vegetation succession. More studies on a landscape scale should continue to provide more insight into the fundamental ecological drivers of the subalpine thicket in the Eastern Carpathians in the face of ongoing climate change.

## Supplementary Information

Supplementary Information.

## Data Availability

Remote sensing raw data and the DTM model are available at: https://data.mendeley.com/datasets/23x8mpfjg9/1.
