# Supplementary material for: Subalpine woody vegetation in the Eastern Carpathians after release from agropastoral pressure
Source: Sci Rep. 2022 Oct 25;12:17897. doi: 10.1038/s41598-022-22248-3 (PMC9596460; doi:10.1038/s41598-022-22248-3)
Supplement: Supplementary file 1 — Supplementary Information. [file 41598_2022_22248_MOESM1_ESM.pdf]

Supplementary Information

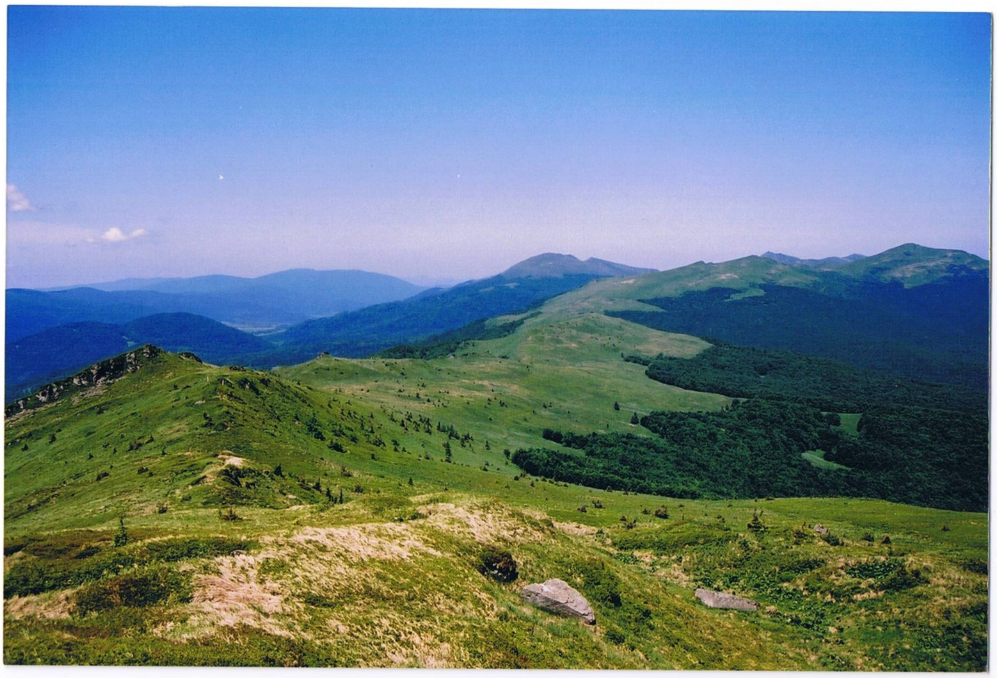

Fig. S1. Bieszczady's landscape: subalpine meadow (*połonina*) and forest zone in the Eastern Carpathian Biosphere Reserve. Bieszczady National Park, part SE, Połonina Bukowska from Kinczyk Bukowski Mt. In background Tarnica Mt. 1346 m a.s.l. Photos. Józef Mitka.

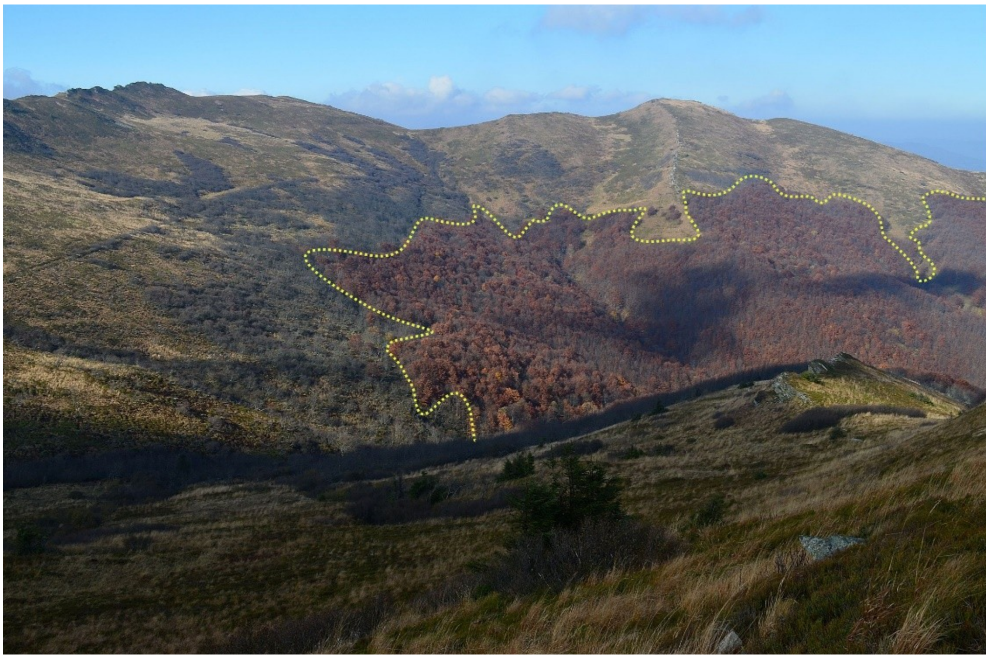

Fig. S2. Upper forest limit in the Bieszczady Mts, NE slope of Krzemień ridge. A border between beech/sycamore-beech forests and subalpine woody vegetation or subalpine meadow. Photos. S. Kucharzyk.

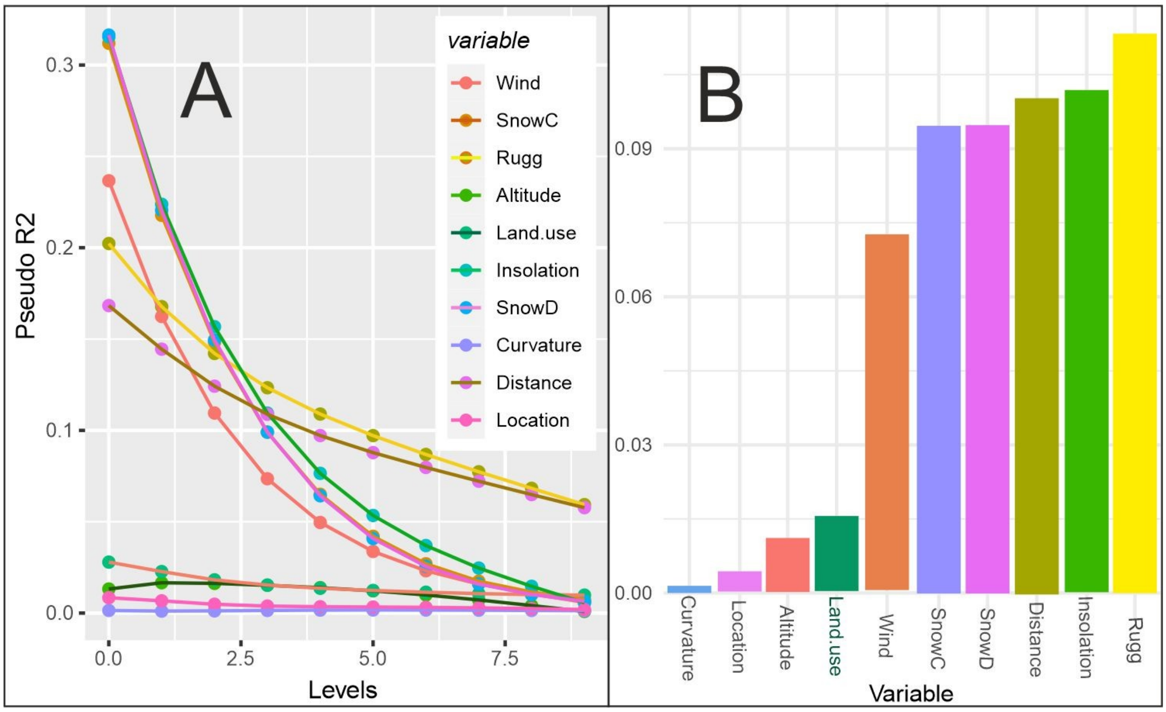

Fig. S3. Dominance analysis of response variables. *(A) Conditional dominance - average additional contribution of the predictors across all model sizes (model size depends on the growing number number of possible combinations of predictors); (B) General dominance – mean value of each predictor's conditonal value. Credit: R Core Team, 2021.*

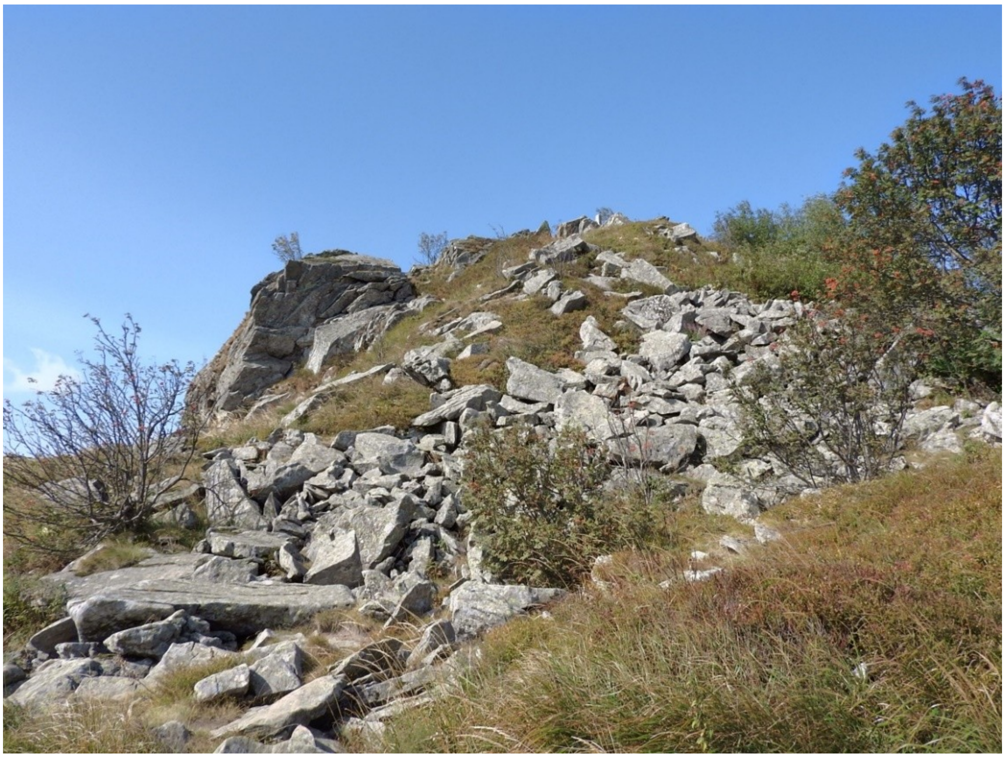

Fig. S4. Topographic irregularities form shelters against unfavorable environmental conditions and the regeneration niche for seedling recruitment and further growth. Rowan and willows among sandstone boulders (grechot). Photos. S. Kucharzyk.

Land use of subalpine meadows

The current range and structure of the vegetation of the mountain pastures for five centuries were shaped by the pastoral economy, but the forestless nature of the highest peaks is considered natural (Kubijowicz, 1926; Winnicki, 1999; see Figs. S1 and S2). Recent archaeological findings from the turn of the Neolithic and early Bronze Age suggest permanent or occasional use of the Bieszczady Mountains by itinerant shepherds from the Corded Ware culture circle of approximately 3 thousand years BC (Pelisiak, 2018). Confirmation of it comes from palynological studies carried out in high mountain peat bogs (Ralska-Jasiewiczowa et al., 2005). In the layer dated after 1090 ± 30 uncal BP, a slight share of pollen of plants considered anthropogenic indicators was found, without the presence of pollen of cereals. The high proportion of green alder and grass (*Poaceae*) pollen, fern spores (*Filicales*), and the increase in pollen of *Betula* and *Salix* suggest that shrubs, herbaceous, and grass plants played a significant role at that time. The upper forest limit was formed by beeches and hazel. The presence of rowan pollen was not found, but it may be due to the biology of this insect-pollinated species. The next separated peatland level indicates a sharp decrease in the share of shrubs and ferns and an increase in anthropogenic indicators, which is probably related to the development of settlement and pastoralism.

The analysis of historical documents confirms the existence of mountain pastures in the mid-15th century, before the intensive colonization of these lands. From the end of the Middle Ages, for the next five hundred years, deforestation of the valley areas, the creation of clearings on the slopes, and the widening of the meadows by lowering the boundary of forests and subalpine thickets (Zarzycki, 1963; Augustyn, 1993; Kucharzyk and Augustyn, 2006)

occurred. These processes were not linear, because three times in the 17th and 18th centuries there were regressions in settlement and the development of secondary forests in abandoned settlements (Augustyn, 2006). Probably that period was related to the increased frequency of green and gray alder pollen visible in the pollen profile (Ralska-Jasiewiczowa et al., 2005). A significant increase in the area of mountain meadows related to the intensification of the pastoral economy occurred in the first half of the 19th century (Augustyn and Kucharzyk, 2012). In the years 1900-1914, the high profitability of cattle breeding resulted in a significant increase in the number of herds grazed in the pastures (over 1.5 heads per 1 ha in 1913). After the First World War, the introduction of grazing fees and the decreasing demand resulted in a gradual reduction in all meadows to the level of 0.7 heads per 1 ha in 1925 (Kubijowicz, 1926; Kucharzyk and Augustyn, 2006). During the economic use of pastures, the occurrence of shrubs was limited by browsing, and partially by mowing. According to the archival cadastral maps, the tax categories of shrubbery and wooded meadows and pastures above the upper forest border took up only about 3% of the total area of the meadows (Kucharzyk and Augustyn, 2006). The floristic works carried out at that time indicated the prevalence of green alder, a typical species of wet shrub communities, significantly decreased by the end of the 19th century (Kotula, 1883).

As a result of the great crisis in the 1930s, many estates completely abandoned the use of hard-to-reach agricultural land. After 1947, as a result of the almost complete depopulation of the Bieszczady area, the economic use of the meadows ceased to be null. Already the first post-war botanical works indicated the development of subalpine thickets formed by green alder and rowan in Halicz, Tarnica, Rozsypaniec, Bukowe Berdo, and on Wielka Rawka peaks in the zone 1100-1250 m above sea level (Zarzycki, 1963; Jasiewicz, 1965). The dominant species was green alder, accompanied by mountain ash, sycamore, and Silesian willow (Zarzycki, 1963).

The photointerpretation of aerial photographs from 1968 reveals that shrub communities already occupied about 10% of the mountain meadows, most of which were loose with 30-50% coverage (Kucharzyk, pers. data). In 1973, most of the mountain meadows were strictly protected within the Bieszczady National Park, sanctioning the ongoing succession processes. According to the research carried out by in 1994, the area of shrubs increased slightly over the next 25 years, while their density increased (Kucharzyk and Augustyn, 2008).

According to historical sources, one of the first mountain pastures on which they were abandoned were Wielka Rawka and Bukowe Berdo, overgrown today to a much greater extent than other ranges (Kucharzyk and Augustyn, 2008; Durak et al., 2015 a and b). According to Durak (2015b), in extensive pastures, the dispersed rowan trees were not eliminated, but only grazed by animals and thus kept in a dwarf form. After the cessation of pastoralism, well-rooted cohorts began to grow relatively quickly. Faster overgrowth of clearings used by the shepherds than mowing ones was also confirmed by the research of Kucharzyk and Augustyn (2010).

LOGIT MODEL OF EXPANSION OF SUBALPINE THICKETS

The probability of a raster cell's overgrowth in subalpine woody vegetation was calculated according to the formula:

$P=1/1+e^{-logit(p)}$

The exponent of the power *logit(p)* takes the form of a generalized linear function *logit (p)* = $\beta_1 x_1 + \ldots + \beta_k x_k$, where x - are successive explanatory variables and β are the regression coefficients for these variables.

According to the results in Table 2, the final equation is as following:

logit(p) = 14.928 - 1.642**Wind* + 0.0123**Snow cover* + 3.895**Rugged*. -0.006**Altitude* - 0.375**Land use 2* - 1.570**Land use 2* - 0.009**Snow distance* - 0.336**Curvature* - 0.000006**Insolation* - 1.290**Distance*" + 3.876**Location 2* + 2.060**Location 3.*

Based on the logit model, the forecast of ovegrowth of the subalpine meadows by the thickets was obtained (the calculations were made for each 20 ×20 grid cell according to the explanatory variables in Table 2). The result is as follows:

Type of cluster	Probability of vegetation overgrowth according to logit model						Total
	0.00-0.49	0.50-0.59	0.60-0.69	0.70-0.79	0.80-0,89	0,90-1,00	
HH	2.74	0.65	0.84	1.16	1.77	9.09	16.25
HL	0.25	0.02	0.04	0.03	0.06	0.16	0.56
LH	0.55	0.09	0.09	0.12	0.1	0.26	1.20
LL	30.39	0.54	0.47	0.34	0.33	0.26	32.33
NN	31.46	2.54	2.57	2.83	3.3	6.96	49.65
Total	65.39	3.83	4.02	4.47	5.56	16.73	100

Table 1. The percentage of 20 × 20 m grid cells with the theoretical probability of overgrowth, concerning the type of clusters according to the Anselin Moran I classification. In the logit model, the explained variable is binary, for the probability lower than 0.5, the variable takes the value 0 (not overgrown), for the probability higher than 0.5, the variable takes the value 1 (overgrown). Thus, the percentage of the NN cells favorable to the overgrown is equal [(49.65-31.46)/49.65] × 100 = 36.6%.

The logistic regression model estimation was elaborated only for HH and LL clusters (hot and cold spots, Table 2). Thus, the percentage of correct probability prediction is an assessment of the goodness of fit of the model and averaged 90.2%. The LL clusters

(explained binary variable "0") overlap with the projected cells without thickets in 94%, and in the case of HH clusters (binary variable "1"), the agreement with the predicted occurrence of cells with thickets is 84%.
